# Transcription factors involved in plant responses to cadmium-induced oxidative stress

**DOI:** 10.3389/fpls.2024.1397289

**Published:** 2024-06-13

**Authors:** Hewan Zhang, Lingli Lu

**Affiliations:** ^1^ Key Laboratory of Environment Remediation and Ecological Health, College of Natural Resource & Environmental Sciences, Zhejiang University, Hangzhou, China; ^2^ Key Laboratory of Agricultural Resource and Environment of Zhejiang Province, College of Environmental and Resource Sciences, Zhejiang University, Hangzhou, China

**Keywords:** cadmium, transcription factor, WRKY, ERF, MYB, bHLH

## Abstract

Cadmium (Cd) is a heavy metal highly toxic to living organisms. Cd pollution of soils has become a serious problem worldwide, posing a severe threat to crop production and human health. When plants are poisoned by Cd, their growth and development are inhibited, chloroplasts are severely damaged, and respiration and photosynthesis are negatively affected. Therefore, elucidating the molecular mechanisms that underlie Cd tolerance in plants is important. Transcription factors can bind to specific plant cis-acting genes. Transcription factors are frequently reported to be involved in various signaling pathways involved in plant growth and development. Their role in the resistance to environmental stress factors, particularly Cd, should not be underestimated. The roles of several transcription factor families in the regulation of plant resistance to Cd stress have been widely demonstrated. In this review, we summarize the mechanisms of five major transcription factor families–WRKY, ERF, MYB, bHLH, and bZIP–in plant resistance to Cd stress to provide useful information for using molecular techniques to solve Cd pollution problems in the future.

## Introduction

Cadmium (Cd),a non-essential toxic heavy metal, occurs naturally and is released into the environment through agricultural and industrial activities (Genchi et al., 2020). Resolving Cd pollution presents significant challenges. Cd infiltrates plant organs and tissues via ectoplasmic and symplastic pathways (Song et al., 2017). Under Cd stress, plant growth and cell division slow, while photosynthesis and transpiration rates varyingly decrease (El Rasafi et al., 2022). Furthermore, Cd accumulated in plants can enter the food chain, affecting animals and humans (Okereafor et al., 2020),increasing risks of cardiovascular diseases, and causing kidney dysfunctions and metabolic disorders (Tinkov et al., 2018; Abougabal et al., 2020; Devereux and Navas-Acien, 2013). Recently, heavy metal accumulation rates in the soil of China have notably exceeded those in other countries. Therefore, urgently addressing this issue is crucial (Yuan et al., 2021). Understanding the Cd accumulation mechanisms in plants will enhance their use in bioremediating Cd-contaminated soils.

Cd is transported via trace element pathways that includes iron, manganese, and zinc. It enters plants through the roots and is present as ions in the cytoplasm, vacuoles, and vascular tissues (Sterckeman and Thomine, 2020). Plant respond to Cd stress through three main mechanisms: (I) chelation, (II) compartmentalization, and (III) plant–nutrient interactions. Firstly, although both metallothionein (MT) and phytochelatin (PC) play roles in reducing Cd toxicity, PC is primarily responsible for mitigating heavy metal toxicity in plants (Grennan, 2011). PC, a small cysteine-rich peptide, bind metals via the sulfhydryl (SH) group. Phytochelatin primarily detoxifies Cd, leveraging the high affinity of Cd^2+^ for sulfur-containing ligands (Seregin and Kozhevnikova, 2023). Studies indicate that Cd stimulates PC biosynthesis in corn, forming PC-Cd complexes that migrate to root vacuoles, thereby reducing Cd toxicity. Additionally, this mechanism prevents root saturation by transferring excess Cd from roots to shoots (Zare et al., 2023). Secondly, to protect vital organs from stress, most plants sequester Cd in vacuoles. For instance, in barley cells, Cd primarily accumulates in vacuoles, facilitated by the stimulating effects of ATP and GSH in the plant (Thomas and Reid, 2021). Finally, appropriate plant nutrition can mitigate Cd damage. Adequate application of Zn fertilizer to plant foliage effectively prevents Cd poisoning in plants and reduces Cd concentrations in wheat seeds harvested from contaminated soils. The protective mechanism likely involves Zn’s crucial role in synthesizing and activating antioxidant enzymes, which diminishes Cd-induced ROS production (Saifullah et al., 2014). Besides the three main mechanisms, plants employ additional strategies to regulate Cd tolerance.

Plant response to Cd stress are closely associated with oxidatives stress and hormone signaling. Cd disrupts the ability of antioxidants in plant to counter oxidative stress due to its high affinity for oxygen, nitrogen, and sulfur atoms. Exogenous NO application enhances antioxidant enzymes activity, limiting Cd absorption and promoting nutrient accumulation in tomato (Ahmad et al., 2018). Together, melatonin and NO enhance Cd tolerance in wheat (Kaya et al., 2019). The interaction between NO and H_2_S significantly improves plant resistance to Cd by enhancing the antioxidant defenses and essential nutrient absorption (Kaya et al., 2020). This interference reduces catalase (CAT) and superoxide dismutase (SOD) activities, leading to lipid peroxidation and oxidative proteins accumulation (Khanna et al., 2022). Phytohormones enhance plant resistance to heavy metals by activating regulatory and signal transduction molecules. Cd stress suppresses gene expression related to IAA biosynthesis and transport (Chen et al., 2023). Applying abscisic acid (ABA) and salicylic acid (SA) increases Cd transport from roots to stems (Zhu et al., 2020a). In Duckweed *Wolffia arrhiza*, ABA levels increase while gibberellic acid (GA) levels decrease following Cd treatment (Chmur et al., 2020).

Studying the molecular mechanisms underlying Cd tolerance in plants is essential for further understanding. *OsMTP1*,a bivalent cation transporter, efficiently translocates Zn, Cd, and other heavy metals, maintaining plant ion homeostasis (Yuan et al., 2012). *StMTP9*, significantly responds to Cd stress, substantially reducing Cd content in yeast cells (Li et al., 2021a). Overexpression of *HaMTP10*, an antitransporter of Cd, reduces Cd accumulation (Li et al., 2023). *BcNRAMP1* overexpression enhances Cd transport and accumulation (Yue et al., 2021). Overexpression of *PcNRAMP1* in *Populus x canescens* increased Cd net flux by 39–52% (Yu et al., 2022). Arabidopsis reduces Cd toxicity by downregulating genes (*AtIRT1*, *AtHMA2*, *AtHMA4*), enhancing Cd uptake and distal translocation (Lei et al., 2020). *ZmHMA3* enhances maize’s tolerance to Cd by facilitating its absorption and transport (Liao et al., 2023). The constitutive expression of *NcZNT1* in *Noccaea caerulescens* roots suggests its role in long-distance metal transport from roots to shoots by aiding Zn/Cd influx into xylem-loading cells (Lin et al., 2016). Upregulation of *BcABCC1/2* expression enhances Cd compartmentalization in root vacuoles, reducing its accumulation in the edible parts of pakchoi (Huang et al., 2021). *OsABCC9* mediates Cd tolerance and accumulation in rice by compartmentalizing Cd into root vacuoles (Yang et al., 2021). Transcription factors play a crucial role in regulating both biological and abiotic processes in plants. A deeper understanding of transcription factors is essential for elucidating the molecular mechanisms of plant Cd tolerance.

This review summarizes the role of transcription factors in plant Cd tolerance. Transcription factors are crucial for plant transcriptional regulation, affecting growth and adaptation to adverse environments. Consequently, transcription factors are pivotal in regulating plant tolerance to heavy metals (Strader et al., 2022). Plant secondary metabolites, synthesized in specific organs or tissues during distinct developmental stages or in response to environmental stimuli, are crucial for plant-environment interactions. Consequently, these genes are transcriptionally regulated by multiple TFs (Yang et al., 2012). Recent studies confirm the involvement of several transcription factor families in plant Cd tolerance mechanisms. This review aims to summarize how the WRKY, ERF, MYB, bHLH, and bZIP transcription factor families regulate Cd tolerance in plants.

## WRKY TFs regulate plant Cd stress responses by modulating the redox system

WRKY TFs belong to a large family of transcriptional regulators that are integral to the signaling network regulating various plant processes. WRKY proteins typically function as both repressors and activators, influencing the repression and activation of crucial plant processes (Rushton et al., 2010). Recent studies indicate that WRKY TFs are crucial in regulating plant defense and disease resistance (Eulgem and Somssich, 2007). The expression of several WRKY genes is strongly and quickly induced by drought and high salinity, suggesting their regulatory roles in these signaling pathways (Chen et al., 2012). Additionally, this expression can be triggered by heavy metals. Recently, many studies have focused on how WRKY TFs regulate Cd tolerance in plants. Most WRKY TFs in plants are localized in the nucleus and predominantly expressed in the roots, though they are present in all plant tissues (**Table 1**).

**Table 1:** Functional analysis of WRKY transcription factor family in different plants.

Plant	Gene	Expression sites	Subcellular location	Mechanism	Downstream gene	Reference
*Arabidopsis thaliana*	*AtWRKY12*	Root, stem, leaves	/	Repressing the expression of phytochelatin synthesis-related genes.	*GSH1*	(Han et al., 2019)
	*AtWRKY13*	Cauline leaf, stem, root, inforescence, silique	Nucleus	Increasing Cd efflux and reducing ROS accumulation	*PDR1, PDR2, PDR8*	(Zhang et al., 2020)
	*AtWRKY13*	/	/	Cd extrusion	*PDR8*	(Sheng et al., 2019)
	*AtWRKY18、40、60*	/	/	Regulate the H_2_S signaling pathway	*LCD, DCD, DCD2, DES, NFS2*	(Liu et al., 2015)
Photo	*StWRKY6*	Root, stem, leaves	Nucleus	Enhance the activity of CAT	*/*	(Guandi et al., 2023)
Pepper	*CaWRKY41*	Root, leaves	Nucleus	Inhibit the expression of ROS-scavenging enzymes	*ZIP3, ZIP5, ZIP9*	(Dang et al., 2019)
Soybean	*GmWRKY142*	Root	Nucleus	Decrease Cd uptake	*ATCDT1, GmCDT1-1, GmCDT1-2*	(Cai et al., 2020)
	*GmWRKY27*	/	/	Lower the levels of endogenous ABA and jasmonic acid	/	(Imran et al., 2022)
	*GmWRKY172*	Leaves, stem, root,flowers	Nucleus	Increase the activity of POD and H_2_O_2_	/	(Xian et al., 2023)
Kenaf	*HcWRKY71*	Leaves, stem, root	/	/	/	(Li et al., 2021b)
Poplar	*PyWRKY75*	Root, leaves, stem	Nucleus	Increase antioxidant enzyme activity	/	(Wu et al., 2022)
	*PyWRKY48*	/	/	Improve the activity of antioxidant enzymes	*PaGRP*, *PaPER* and *PaPHOS*	(Wu et al., 2023a)
Wheat	*TaWRKY22*	/	/	Activate ROS-scavenging system	*TaCOPT3D*	(Liu et al., 2022a)
	*TaWRKY70*	Root, shoot	Nucleus	Reduce the Cd transfer coefficient	*TaCAT5*	(Jia et al., 2021)
	*TaWRKY74*	Root, shoot	Nucleus/cytoplasm	Affect the expression of ASA-GSH synthesis genes and suppressing the expression of Cd transporter genes	/	(Li et al., 2022)
*Sedum alfredii*	*SaWRKY7*	Root, stem, leaves	Nucleus	/	/	(Wang et al., 2019)
*Tamarix hispida*	*ThWRKY7*	Root, leaves	/	Regulate ROS homeostasis	*ThVHAc1*	(Yang et al., 2016)
Maize	*ZmWRKY4*	Leaves	Nucleus/cytoplasm	Increase antioxidant enzyme activity	*ZmSOD4* and *ZmcAPX*	(Hong et al., 2017)
	*ZmWRKY64*	Root,leaves	Nucleus	Regulate ROS homeostasis	*ZmSRG7*	(Gu et al., 2024)
Rice	*OsWRKY71*	Leaves, meristem, root, callus, panicle, pollen, stigma, stem, seed	/	/	/	(Paul and Roychoudhury, 2018)
Sugarcane	*ScWRKY6*	Root, bud, leaves, stem pith, epidermis	Nucleus	/	/	(Zhang et al., 2018)

Cd tolerance mediated by WRKY TFs has been demonstrated across various model plants and food crops. in *Arabidopsis thaliana*, *AtWRKY12* indirectly suppresses genes related to PC synthesis and negatively impacts Cd accumulation and tolerance (Han et al., 2019). Conversely, *AtWRKY13* employs distinct mechanisms, enhancing Cd tolerance in plants by influencing H_2_S synthesis (Sheng et al., 2019; Zhang et al., 2020). Similarly, multiple studies have shown that WRKY transcription factors regulate H_2_S to affect Cd tolerance. Plants with double or triple mutations in *WRKY18, WRKY40*, and *WRKY60* produced H_2_S at a higher rate under Cd stress than wild-type and single-mutant plants, suggesting increased Cd resistance. *WRKY18*, *WRKY40*, and *WRKY60* primarily function as repressors, regulating genes that encode H_2_S synthase. These findings indicate that WRKY transcription factors regulate the H_2_S signaling pathway, aiding plant resilience to Cd stress (Liu et al., 2015). Besides H_2_S, WRKY transcription factors also regulate antioxidant enzyme activity and synthesis; overexpressing *TaCOPT3D* in wheat decreases ROS levels and enhances antioxidant enzyme activity under Cd stress. Additionally, the transcription factor *TaWRKY22* targets the *TaCOPT3D* promoter, playing a role in its regulation under Cd stress. These findings highlight *TaCOPT3D*’s crucial role in plant adaptation to Cd stress through its interaction with *TaWRKY2* (Liu et al., 2022a). Expression of *TaWRKY70* in *Arabidopsis* enhances growth and biomass production under Cd stress. Moreover, *Arabidopsis* overexpressing *TaWRKY70* exhibit increased SOD, POD, and CAT activities under Cd stress compared with wild-type plants. Thus, the improved growth of transgenic *Arabidopsis* under Cd stress is due to enhanced antioxidant capacity and reduced oxidative damage (Jia et al., 2021). *TaWRKY74* consistently mitigates Cd toxicity in wheat by influencing ASA-GSH synthesis genes and repressing Cd transporter genes (Li et al., 2022). In rice, *OsWRKY71* significantly induces Cd stress tolerance in leaves, with transcript levels peaking 24 hours post-stress initiation (Paul and Roychoudhury, 2018).

Beyond model plants and food crops, the role of WRKY TFs in Cd tolerance has been explored in various other plants. In *Sedum alfredii*, *SaWRKY7* responds to Cd induction, potentially playing a crucial role in Cd accumulation and tolerance (Wang et al., 2019). In *Tamarix hispida*, *ThVHAc1* and *ThWRKY7* likely jointly regulate Cd tolerance, with *ThWRKY7* potentially control *ThVHAc1* to boost abiotic stress tolerance (Yang et al., 2016). Some WRKY TFs increase *Zea mays* tolerance to Cd by promoting GSH synthesis *in vivo* (Mei et al., 2017). *ZmWRKY64* improves Cd tolerance by upregulating *ZmSRG7* transcription, thus maintaining ROS homeostasis (Gu et al., 2024). Cd upregulates of the *ZmWRKY4* expression enhances antioxidant enzyme activity (Hong, Cheng et al., 2017). Similarly, a positive feedback loop between H_2_O_2_ accumulation and *CaWRKY41* upregulation coordinates pepper plants’ responses to Cd exposure. This mechanism might reduce Cd tolerance by enhancing Cd uptake through Zn transporters (Dang et al., 2019). In soybeans, *GmWRKY142* directly targets *ATCDT1*, *GmCDT1–1*, and *GmCDT1–2*, reducing plant Cd uptake and enhancing Cd tolerance (Cai et al., 2020). The expression of *GmWRKY27* was slightly upregulated under Cd stress compared with that in untreated plants, suggesting that *GmWRKY27* may play an important role in Cd tolerance in soybeans (Imran et al., 2022). *GmWRKY172* promotes H_2_O_2_ accumulation, increases peroxidase (POD) activity, and reduces Cd transport from roots to stems and seeds (Xian et al., 2023). In Kenaf, *HcWRKY71* expression decreases as CdCl_2_ concentrations increase. The most significant change in *HcWRKY71* expression occurred in the roots under CdCl_2_ stress. Thus, *HcWRKY71* may enhance Kenaf’s Cd tolerance by regulating root gene expression during stress (Li et al., 2021b). In poplars, overexpression of *PyWRKY75* increases chlorophyll content and promotes growth under Cd stress, and correlates with increased tolerance due to the protective effects of antioxidant enzymes (POD, SOD, CAT, APX) and osmoregulatory substances (soluble sugars). Overexpression of *PyWRKY75* also increases ASA, GSH, and PCs content in poplars, potentially promoting Cd accumulation. Therefore, *PyWRKY75* may serve a positive regulatory role under Cd stress in poplars, suggesting its potential as a candidate gene for plant Cd tolerance (Wu et al., 2022). *PyWRKY48* enhances the Cd tolerance by upregulating the expression of PaGRP, PaPER and PaPHOS, which encode antioxidase-related proteins (Wu et al., 2023a). In sugarcane, CdCl_2_ induces the expression of *ScWRKY6*, *ScWRKY6* likely contributes to sugarcane’s response to Cd stress by promoting stress protein and proline accumulation and enhancing antioxidant enzyme activity (Zhang et al., 2018). *StWRKY6* increases CAT activity, enhancing ROS clearance and reducing oxidative stress in photosynthetic tissues (Guandi et al., 2023).

Transcriptome data reveal that the expression level of WRKY TFs changed in different plants in response to Cd stress. For example, In *Sedum plumbizincicola*, 7 *SpWRKYs* were significantly upregulated by Cd stress (Wang et al., 2023). The expression level of seven genes (*HvWRKY1, 3, 13, 15, 24, 43*, and *55*) increased by more than two-fold in barley after CdCl_2_ treatment. The expression levels of *HvWRKY3* and *HvWRKY24* were 20-fold and 10-fold higher, respectively, than those in controls (Zheng et al., 2021). In particular, the expression of *HvWRKY32* and *HvWRKY80* was significantly decreased. Conversely, *BnaWRKY49* and *BnaWRKY38* were upregulated after Cd treatment, and *BnaWRKY7*, *27*, *38*, *56*, and *60* were identified as key Cd-related genes (Ma et al., 2022). In creeping bentgrass, the expression of *WRKY33*, *WRKY12*, *WRKY2*, *WRKY23*, *WRKY75*, and *WRKY53* was either upregulated or downregulated after Cd treatment (Yuan et al., 2018). The expression of 11 WRKY genes increased with increasing Cd content in the leaves of golden raintrees (He et al., 2021). In hemp, the expression of CsWRKY genes changed significantly under Cd stress, with 14 of the 40 predicted genes responding to Cd stress. In particular, the expression levels of these three genes increase by more than 10-fold in response to Cd stress (Xin et al., 2016). Finally, the expression of 22 rice WRKY TFs was upregulated under Cd stress (Tan et al., 2017).

In summary, the WRKY TFs modulates Cd tolerance in plants through the redox system, specifically influencing the synthesis and activity of antioxidant enzymes (POD,SOD,CAT and APX) and redox-related substances (ROS,H_2_S,NO,GSH).Currently, transcriptome analysis. Currently, transcriptome analyses indicate that WRKY factors respond to Cd stress. While specific signaling pathways remain unidentified, these studies guide future research directions.

## ERF TFs regulate plant Cd stress responses by modulating ROS, nitrate, and ethylene synthesis and activity

The ERF TFs regulates stress responses linked to ethylene, a significant stress hormone induced by various abiotic factors (Wang et al., 2022a). ERF TFs were initially identified due to their ability to bind to promoters of stress response genes. Research has shown that besides responding to stress hormones like ethylene, jasmonic acid, and abscisic acid, ERF TFs are also triggered by various biotic and abiotic stresses such as salinity, osmotic stress, drought, hypoxia, temperature fluctuations, and pathogen infections (Licausi et al., 2013). Similarly, ERF TFs are induced by cadmium and play crucial roles in regulating plant Cd tolerance. Most plant ERF TFs are localized in the nucleus and expressed across all tissues, predominantly in the roots. (**Table 2**).

**Table 2:** Functional analysis of ERF transcription factor family in different plants.

Plant	Gene	Expression sites	Subcellular location	Mechanism	Downstream gene	Reference
*Arabidopsis thaliana*	*AtERF34/AtERF35*	/	/		*NRT1.8*	(Xie et al., 2021)
	*AtERF1B/AtERF104*	/	/	Promote nitrate assimilation and photosynthesis	*NRT1.8、NRT1.5*	(Zhang et al., 2014)
Wheat	*TaMYC8/TaERF6*	Root, shoot	Nucleus	Affect the ethylene biosynthetic pathway	/	(Wang et al., 2022b)
	*TdSHN1*	Seedlings	/	Detoxification of ROS	/	(Djemal and Khoudi, 2022)
Pepper	*CaPF1*	/	/	/	/	(Jung Won et al., 2008)
Barley	*HvRAF*	/	/	/	/	(Jung and Kim, 2007)
Rice grain	*OsERF141*	/	/	/	/	(Ailing et al., 2020)
Bean	*PvERF15*	Nucleus	Nucleus	/	*PvMTF-1*	(Lin et al., 2017)
*Glycyrrhiza uralensis Fisch*	*ERF061*	Root	/	Stimulate ROS-related gene expression	/	(Chahel et al., 2020)
*Pinus virginiana Mill*	*CaPF1*	/	/	Increase antioxidant enzyme activity	/	(Tang et al., 2005)
*Aquilaria sinensis*	*AsERF1*	Root, stem, leaves	Nucleus	/	/	(Li et al., 2020)

Cd tolerance mediated by ERF TFs has been demonstrated in various model plants and food crops. For example, in *Arabidopsis*, the mRNA level of *NRT1.8*, a nitrate transporter inducible by Cd, are significantly upregulated in the root system following Cd exposure, Specifically, *ERF34* and *ERF35* bind to the *NRT1.8* promoter, regulating Cd tolerance (Xie et al., 2021). Similarly, *ERF1B* and *ERF104* bind directly to the *NRT1.8* promoter, mediating its expression under Cd induction, which is crucial for understanding plant responses to Cd stress (Zhang et al., 2014). In wheat, *TaMYC8* directly regulates *TaERF6* expression and negatively affects Cd tolerance by activating *TaERF6* transcription via binding to its promoter (Wang et al., 2022b). *TdSHN1*, a SHINE-type ERF transcription factor, was significantly upregulated following Cd induction. After *TdSHN1* knockout, transgenic tobacco exhibited longer roots, increased biomass, higher chlorophyll content, and reduced ROS production compared to wild-type under heavy metal stress. Additionally, transgenic tobacco displayed increased activity of ROS-scavenging enzymes, SOD and CAT (Djemal and Khoudi, 2022). In barley, the ERF-type transcription factor *HvRAF*, particularly its C-terminal region when overexpressed, enhances yeast Cd toxicity tolerance (Jung and Kim, 2007). A study on rice’s interaction network revealed that the *OsERF141*, encoding a transcription factor, is a potential target for specific miRNAs. Interestingly, *osa-miR5493* expression negatively correlates with *OsERF141*, suggesting *osa-miR549*3’s potential role in regulating Cd stress through *OsERF141* expression (Ailing et al., 2020). *PvERF15* in soybean (Phaseolus vulgaris) directly binds to the *PvMTF-1* promoter and is strongly induced under Cd stress. Knocking down *PvERF15* reduces Cd tolerance, positioning *PvERF15* as a key element of the Cd stress transcriptional pathway with *PvMTF-1* as a downstream target (Lin et al., 2017).

In pepper, *CaPF1*, an ERF/AP2-type TF, regulates plant tolerance to Cd by regulating the oxidative stress mechanism (Jung Won et al., 2008). In *Pinus virginiana* Mil, *CaPF1* overexpression in transgenic plants resulted in greater Cd tolerance than in the control, presumably by regulating antioxidant enzyme activity and lipid peroxidation under Cd stress (Tang et al., 2005). Furthermore, the relative expression of *LrERF061* significantly increased in *Glycyrrhiza uralensis* Fisch under Cd stress. The activity of antioxidant enzymes (SOD, CAT, and POD) was enhanced in transgenic plants overexpressing *LrERF061* at high Cd concentrations (Chahel et al., 2020). In *Aquilaria sinensis*, the expression level of *AsERF1* increased under heavy metal stress, reaching 3.5 times the level of the control group at the highest point. These results indicate that heavy metal stress induces the expression of the *AsERF1* encoding gene (Li et al., 2020).

Current transcriptome data show that ERF TFs positively responds to Cd stress. Based on transcriptome analysis, 15 StAP2/ERF genes were randomly selected from 181 potato genes to test their involvement in the plant response to Cd stress. The results showed that most genes were sensitive to Cd stress. Particularly, in the leaves, *StAP2/ERF027*-, *StAP2/ERF140*, and *StAP2/ERF164* encoding genes were significantly upregulated and showed maximum expression levels 24 h after Cd treatment. The yeast complementation assays showed that *StAP2/ERF129* and *StAP2/ERF139* (subgroup 1) promoted Cd accumulation (Cd-reduced), whereas *StAP2/ERF044* and *StAP2/ERF180* (subgroup 2) promoted Cd accumulation and inhibited growth (Cd-accumulated). In turn, the *StAP2/ERF075*, *StAP2/ERF077*, and *StAP2/ERF126* encoding genes (subgroup 3) promote Cd accumulation and yeast growth (Cd detoxification type) (Tian et al., 2022). In eggplant, seven genes (*SmERF9, SmERF30, SmERF59, SmERF61, SmERF85, SmERF86*, and *SmERF100*) showed significantly up-regulated expression levels under Cd treatment and a down-regulation trend under prolonged treatment conditions, whereas four genes (*SmERF2, SmERF39, SmERF55*, and *SmERF57*) were consistently down-regulated (Shao et al., 2015). Differentially expressed genes in diploid poplar (*Populus simonii* × P) were identified experimentally. ERFs and differentially expressed genes exhibited similar expression patterns under salinity (NaCl), chlorine (KCl), heavy metal (CdCl_2_), and drought stress. ERF76-overexpressing transgenic poplar was superior to the wild-type in terms of morphological and physicochemical traits, indicating that the ERF76 encoding gene played a significant role in stress resistance (Yao et al., 2017). In *Lycium chinense*, *LchERF* transgenic plants were more tolerant to Cd stress than non-transgenic plants. Overexpression of *LchERF* enhanced Cd tolerance in transgenic tobacco, which may be due to the effect of *LchERF* on tobacco glutathione synthesis-related genes. Under Cd stress, non-transgenic plants consistently accumulate more ROS than those overexpressing *LchERF* (Guan et al., 2015). Transgenic tobacco overexpressing LcMKK showed greater tolerance to Cd stress with enhanced expression of *NtERF* and *NtGSH1*, indicating that LcMKK is associated with enhanced expression levels of ERF synthesis-related genes in tobacco. Evidence suggests that under Cd stress, *NtERF* may be involved in the accumulation of GSH in the plant body, which in turn reduces the toxic effects of Cd on plants (Guan et al., 2016). Finally, in creeping bentgrass, most genes are upregulated, whereas *ERF115* and *ERF4* are slightly downregulated under Cd stress (Yuan et al., 2018). Collectively, these results provide a useful reference for further studies on the relationship between members of the ERF TF family and Cd tolerance in plants.

Our findings indicate that ERF TFs actively respond to Cd stress in plants and play a crucial role in regulating Cd tolerance. The mechanisms through which ERF TFs regulate Cd tolerance involve controlling ROS, nitrate, and ethylene synthesis and activity.

## MYB TFs regulate plant Cd stress responses by influencing antioxidant enzymes and compartmentalization

The myeloblastosis oncogene (MYB) family, a large group of TF, is prevalent in eukaryotes and widely expressed in most plants (Erpen et al., 2018). Previous research has shown that several MYB TFs regulate plant responses to drought and participate in the ABA signaling network (Cominelli et al., 2005; Lee et al., 2020). Plant MYB proteins are variably regulated by ABA, enhancing abiotic stress tolerance. Additionally, MYB TFs react to Cd stress mechanisms in plants and regulate Cd resistance. Most MYB TFs are localized in the nucleus and expressed in various tissues of the plant, although mainly in the root (**Table 3**).

**Table 3:** Functional analysis of MYB transcription factor family in different plants.

Plant	Gene	Expression sites	Subcellular location	Mechanism	Downstream gene	Reference
*Arabidopsis thaliana*	*AtMYB49*	Cotyledons, shoots, young leaves, flowers, siliques, stems	Nucleus	Affect the synthesis of ABA	/	(Zhang et al., 2019)
	*AtMYB59*	Root, leaves	/	Negatively regulate Ca homeostasis and signaling	*CMLs, KIC, CAX1, ACA1*	(Fasani et al., 2019)
	*AtMYB75*	/	/	Anthocyanin-mediated reactive oxygen species homeostasis and PC content	*ACBP2, ABCC2*	(Zheng et al., 2022)
	*AtMYB4*	/	/	Coordinate activity of improved antioxidant defense system	*PCS1, MT1C*	(Agarwal et al., 2020)
Rice	*OsMYB34*	/	Nucleus	/	/	(Shalmani et al., 2021)
	*OsMYB36*	Root, stems, leaf blades, leaf sheath	Nucleus	Interfere with Casparian strip formation in the endodermis, distinct effects on the selective uptake of mineral elements in roots	*OsCASP1, OsESB1*	(Wang et al., 2022b)
	*OsMYB45*	Leaves, husks, stamens, pistils, lateral roots	Nucleus	Decrease CAT activity	/	(Hu et al., 2017)
Walnut	*JrMYB2*	Root, leaves	/	/	/	(Xu et al., 2018)
*Salicornia brachiataRoxb*	*SbMYB15*	/	/	Increase antioxidant enzyme activity	/	(Sapara et al., 2019)
Ramie	*BnMYB2*	Root, stem, leaves	Nucleus, cytosolic	/	/	(Zhu et al., 2020b)
	*BnMYB1*	Root, stem, leaves	/	/	/	(Zhu et al., 2018)
	*BnMYB3*	Root, stem, leaves		/	/	(Zhu and Shi, 2019)
Tomato	*MYB1*	/	/	/	/	(Hasan et al., 2016)
Soybean	*MYBZ2*	/	/	/	/	(Chmielowska-Bak et al., 2017)
*Potentilla sericea*	*PsMYB62*	Root, stem, leaves	/	Enhance the activities of SOD,POD and CAT	/	(Feng et al., 2023)
*Populus euphratica*	*PeRAX2*	Root, leaves	/	Induce the synthesis of H_2_O_2_ and improve the activities of CAT, SOD and POD	*AtANN1*	(Yan et al., 2024)

Members of the MYB TF family play significant roles in plant Cd tolerance mechanisms. For example, in *Arabidopsis*, *myb49* overexpression results in a significant increase in Cd accumulation, which is significantly reduced by the knockdown of *myb49*. Further studies have shown that *myb49* positively regulates the expression of *bHLH38* and *bHLH101* by directly binding to their promoters, leading to the activation of iron-regulated *TRANSPORTER1*, which encodes a metal transporter protein associated with Cd uptake. *myb49* also binds to the heavy metal-associated plant heteropropenylated protein (*HIPP22*) and *HIPP44* promoter regions, leading to their upregulation and subsequent Cd accumulation. Therefore, *AtMYB49* regulates Cd accumulation by directly regulating the expression of *bHLH38*, *bHLH101*, *HIPP22*, and *HIPP44* (Zhang et al., 2019). Furthermore, the germination rate of the *Arabidopsis MYB59* knockout mutant was significantly higher than that of the wild type or *MYB59* overexpressing mutant in the Cd-containing medium. This suggests that *AtMYB59* may be involved in Cd stress resistance in *Arabidopsis* (Fasani et al., 2019). MYB are crucial for the compartmentalization of Cd.*AtMYB75* likely serves as a positive regulator of Cd tolerance by targeting *Arabidopsis* stress-related genes *ACBP2* and *ABCC2*, which are involved in regulating ATP for Cd compartmentalization in vacuoles (Zheng et al., 2022). They regulate the ATP required for Cd to be compartmentalized by vacuoles. *AtMYB4* boosts plant antioxidant activity by engaging in the glutathione pathway, activating and regulating the expression of *PCS1* and *MT1C* in response to Cd stress. This regulation aids in the chelation of Cd by PC and MT, allowing the complexes to be compartmentalized in vacuoles (Agarwal et al., 2020). In rice, *OsTAZ4* promotes plant resistance to Cd stress by regulating ROS endostasis; furthermore, *OsTAZ4* can promote plant resistance to multiple heavy metals by interacting with the *OsMYB34* transcription factor (Shalmani et al., 2021). The reduction in Cd accumulation in the knockout *OsMYB36* mutant may be related to the altered abundance of *OsNramp5* mRNA or *OsNramp5* protein, indirectly suggesting that *OsMYB36* plays an important role in Cd tolerance in rice (Wang et al., 2022b). The transgenic rice knockout O*sMYB45* mutant was highly sensitive to Cd treatment, and Cd tolerance was greatly reduced, proving that O*sMYB45* plays an important role in rice resistance to Cd stress (Hu et al., 2017).

MYB reduces the toxicity of Cd by regulating the redox system. In walnuts, *JrVHAG1* acts as a regulator of the Cd stress response by participating in the MYB transcriptional regulatory network by regulating the expression of the *JrMYB2* TF, showing that this TF also plays an important role in the regulation of Cd tolerance in walnuts (Xu et al., 2018). In *Salicornia brachiate* Roxb, the expression of *SbMYB15* increased five-fold under Cd stress. In contrast to the wild type, *SbMYB15* overexpressing transgenic tobacco showed lower Cd uptake and increased growth and chlorophyll content. Furthermore, transgenic tobacco showed higher scavenging activities of antioxidant enzymes (CAT and SOD) and higher transcript levels of the related antioxidant genes *CAT1* and *MnSOD* (Sapara et al., 2019). In ramie, the expression of *BnMYB1* was elevated under Cd treatment and showed a significant and progressive increase with increasing Cd concentrations, suggesting that *BnMYB1* may be involved in the mechanism of ramie resistance to Cd tolerance (Zhu et al., 2018). A significant increase in Cd tolerance and accumulation was observed in *BnMYB2* overexpressing transgenic *Arabidopsis* plants. Thus, *BnMYB2* regulates Cd tolerance and accumulation (Zhu et al., 2020b). Additionally, *BnMYB3* responds positively to Cd stress and is a candidate gene for enhancing Cd tolerance in ramie, with increased expression under Cd stress (Zhu and Shi, 2019). In tomatoes, the GSH-induced enhancement of Cd tolerance is closely associated with the upregulation of the transcription factor *MYB1* and some stress-responsive genes (Hasan et al., 2016). In soybean, *MYBZ2* regulates Cd tolerance by scavenging the ROS generated by NADPH oxidase (Chmielowska-Bak et al., 2017). Overexpression of *PsMYB62* in *Potentilla sericea* enhances the activities of SOD,POD and CAT, resulting in increased H_2_O_2_ content in transgenic plants (Feng et al., 2023). *PeRAX2*, a MYB TF in *Populus euphratica*, regulates the transcription of *AtANN1*, mediates Cd^2+^ absorption via calcium channels, induces H_2_O_2_ synthesis, and enhances CAT, SOD, and POD activities (Yan et al., 2024).

Using transcriptome analysis, the TFs *MYB4*, *MYB10*, *MYB72*, and *MYB107* were screened in *Arabidopsis* after Cd treatment. Owing to their high expression levels, these TFs can be used as candidate genes to study Cd tolerance in this model plant (De Mortel et al., 2008). Similarly, in rice, *OsMYB36* expression was significantly upregulated under Cd stress, whereas the genes encoding *OsMYB3*, *OsMYB7*, *OsMYB15*, *OsMYB23*, *OsMYB47*, *OsMYB76*, *OsMYB89*, and *OsMYB93* were significantly downregulated. However, the expression levels of *OsMYB2*, *OsMYB90*, and *OsMYB99* were not significantly altered (Kang et al., 2022). In hemp, most MYB TF family members respond to Cd stress; specifically, the expression of *CsMYB016*, *CsMYB067*, *CsMYB098*, *CsMYB045*, and *CsMYB024* is downregulated, whereas that of *CsMYB028* is upregulated (Yin et al., 2022). In pepper, 10 CaMYB gene members showed a significant increase in expression in response to Cd stress (Xie et al., 2022). Similarly, RNA-seq results have shown that *MYB4*, *MYB39*, *MYB108*, and *MYB305* are upregulated in creeping bentgrass under Cd stress (Yuan et al., 2018). Furthermore, the expression of the seven MYB genes gradually decreases with increasing Cd content (He et al., 2021). Meanwhile, in the leaves, the myb-like genes *Unigene0019400* and *Unigene0027656* were significantly expressed in the absence of Cd stress but were gradually downregulated with increasing Cd concentration. In turn, the expression of most MYB transcription factors in leaves of wild paper mulberry showed a V-shaped expression trend over a period of 120 h of Cd treatment (Xu et al., 2019). In *Apocynum venetum*, *AvMYB4* and *AvMYB10* are downregulated under Cd stress, whereas *AvMYB8*, *AvMYB11*, *AvMYB48*, and *AvMYB97* are upregulated, indicating that they are candidate genes that respond to Cd stress (Abubakar et al., 2022). In *Ipomoea aquatica*, the transcript expression levels of *IaMYB47*, *IaMYB86*, *IaMYB142*, *IaMYB157*, and *IaMYB147* were upregulated, whereas those of *IaMYB7*, *IaMYB110*, *IaMYB121*, *IaMYB173*, and *IaMYB176* were downregulated after Cd treatment (Liu et al., 2022b). Finally, in barley, the transcript expression and m6A modification levels of MYB TF family members were significantly upregulated under Cd stress (Su et al., 2022).

These results demonstrate that the MYB transcription factor family plays a crucial role in plant Cd tolerance by regulating the synthesis and activity of antioxidant enzymes and interacting with genes related to Cd compartmentalization.

## bHLH TFs regulate plant Cd stress responses by increasing the accumulation of nicotinamide

The basic helix-loop-helix (bHLH) family is a large group of plant transcription factors that function as homodimers or heterodimers to regulate gene expression. Several TFs play unique roles in regulating iron homeostasis in plants (Gao et al., 2019). Recent studies demonstrate that bHLH TFs effectively regulate Cd stress response mechanisms in plants. Subcellular localization analysis shows that most bHLH TFs are located in the nucleus and are predominantly expressed in the root system, though they are found throughout plant tissues (**Table 4**).

**Table 4:** Functional analysis of bHLH transcription factor family in different plants.

Plant	Gene	Expression sites	Subcellular location	Mechanism	Downstream gene	Reference
*Arabidopis thaliana*	*AtbHLH38/AtbHLH39*	Shoot, root	/	Maintain high iron levels through iron transport and the accumulation of nicotinic amine.	/	(Wu et al., 2012)
	*AtbHLH104*	Shoot, root	/	Positively regulate four heavy metal detoxification-associated genes	/	(Yao et al., 2018)
	*AtbHLH39/AtbHLH104*	Shoot, root	/	Increase iron accumulation	/	(Meng et al., 2022)
	*AtbHLH100*	/	/	/	/	(De Mortel et al., 2008)
*Broussonetia papyrifera*	*BpbHLH149*	Root	/	/	/	(He et al., 2022)
Wheat	*TabHLH094*	Shoot, root	Nucleus	Inhibition of ethylene synthesis	*TaMYC8*	(Du et al., 2023)
Soybean	*GmbHLH041*	/	/	/	/	(Liu et al., 2018)
	*ORG3*	Root	Nucleus	Increase the translocation ratio of iron but reduce Cd translocation from the roots to shoots	/	(Xu et al., 2017)

Functional verification has confirmed that bHLH TFs are involved in Cd tolerance mechanisms in both model plants and food crops. The regulation of Cd tolerance by bHLH is related to nicotinamide synthase(NAS).For example, in *Arabidopsis*, gene expression analysis showed that *FIT*, *AtbHLH38*, and *AtbHLH39* expression was upregulated in the root systems of Cd-treated plants, and co-expression of all three activated the expression of *HMA3*, *MTP3*, *IRT2*, and *IREG2*, all of which are associated with heavy metal detoxification. Additionally, co-overexpression enhanced the expression of *NAS1* and *NAS2*, leading to the accumulation of nicotinamide, a key chelator of iron transport and homeostasis. Maintaining high Fe content under Cd stress alleviates Cd toxicity (Wu et al., 2012). In particular, *bHLH104* plays an important role in Cd transport from roots to stems. Evidence shows that *bHLH104* positively regulates four heavy metal detoxification-related genes, namely *IREG2, MTP3, HMA3*, and *NAS4*, which are Cd tolerant genes. This shows that *bHLH104* plays an important role in the regulation of Cd-stress plant responses (Yao et al., 2018). Furthermore, *bHLH39* and *bHLH104* interact functionally with *IMA1* and *IMA3* to enhance Fe accumulation in plants by inducing the expression of Fe transporter genes, resulting in enhanced Cd tolerance (Meng et al., 2022). The transcription factor *bHLH100* is highly expressed under high Cd concentrations and may be a candidate gene for enhancing Cd tolerance (De Morte et al., 2008).In wheat, *TabHLH094* mediates Cd tolerance by modulating *TaMYC8* transcriptional activity and reducing ethylene production (Du et al., 2023).

Beyond model plants and major food crops, molecular mechanisms in various plants show that bHLH TFs respond to Cd stress. In *Broussonetia papyrifera*, *BpabHLH149* not only responds to Cd stress but also enhances yeast resistance, potentially making it a key candidate gene for stress responses in *Caladenia tessellata* (He et al., 2022). In soybean, the expression of *GmbHLH041* increases very significantly after 24 h of Cd stress and is an important candidate gene for screening Cd stress tolerance (Liu et al., 2018). The bHLH OBP3-responsive gene (*GmORG3*) was significantly induced by Cd stress in soybean, and *GmORG3* overexpressing plants increased the rate of iron translocation from the roots to stems while decreasing that of Cd. This suggests that *GmORG3* enhances Cd tolerance by stabilizing iron homeostasis (Xu et al., 2017). In *Noccaea caerulescens*, genes encoding the TFs *bHLH038*, *bHLH039*, *bHLH100*, and *bHLH101* were significantly upregulated under Cd stress, and it is highly likely that they enhance Cd tolerance by maintaining photosynthetic activity (Halimaa et al., 2019).

Current transcriptome data show that bHLH transcription factors positively respond to Cd stress, leading to either up-regulation or down-regulation of their expression. Transcriptome analysis in rice has revealed significant involvement of bHLH TFs in regulating Cd and Zn homeostasis (Adil et al., 2022). Similarly, in *Populus simonii × P. nigra* cross, 69 bHLH family members responded to Cd stress, of which 29 were upregulated, and 40 were downregulated (Yao et al., 2019). Furthermore, in wild *paper mulberry*, the expression of most bHLHs was upregulated in wheat leaves under Cd stress (Xu et al., 2019). Consistently, *bHLH29*, *bHLH38*, and *bHLH47* are upregulated in wheat under Cd stress (Sabella et al., 2021). The expression of bHLHs may be regulated through NA synthesis and maintenance of iron homeostasis in the plant, thereby increasing Cd tolerance (Aprile et al., 2018).

In summary, bHLH TFs primarily regulate Cd transport in plants, likely by binding to and regulating the expression of ion channel-related genes. Additionally, some studies indicate that bHLH enhances the accumulation of nicotinamide, which promotes iron transport. Maintaining high iron levels under Cd stress may alleviate Cd toxicity.

## bZIP TFs influence the long-distance transport of Cd by modulating compartmentalization and the redox system

Basic (region) leucine zipper (bZIP) TFs are characterized by a basic DNA-binding region and an adjacent leucine zipper that facilitates dimerization (Droge-Laser et al., 2018). bZIP proteins occur in various plants and participate in diverse biological processes, such as seed maturation, flower development, plant senescence, and light signaling. bZIP TFs also exert negative regulation on plant growth and development (Zhang et al., 2011). Subcellular localization analysis indicates that bZIPs are predominantly located in the nuclei of plant cells, with varying expression across different plant tissues. In many plants, bZIP TFs are mainly expressed in the roots, with fewer expressed in the stems or leaves (**Table 5**).

**Table 5:** Functional analysis of bZIP transcription factor family in different plants.

Plant	Gene	Expressionsites	Subcellular Location	Mechanism	Downstream gene	Reference
*Arabidopis thaliana*	*AtbZIP30*	/	/	Regulate the expression of glutathione S-transferase gene	*AtGST11*	(Kouno and Ezaki, 2013)
	*AtbZIP44*	Root, shoot	/	Promote compartmentalization	*AtMAN7*	(Wu et al., 2023b)
Ramie	*BnbZIP2*	Root, leaves, stem, shoots tip, female and male flower	Nucleus/cytoplasm	/	/	(Huang et al., 2016)
*Sedum plumbizincicola*	*SpbZIP60*	Root, shoot, leaves	Nucleus	Inhibit ROS accumulation	/	(Lu et al., 2022)
*Broussonetia papyrifera*	*BpbZIP1*	Root, shoot, leaves	/	/	/	(Chen et al., 2022)
*Nicotiana tabacum*	*NtbZIP60*	/	/	BiP alleviate endoplasmic reticulum stress and delay the appearance of Cd^2+^-induced programmed cell death	/	(Xu et al., 2013)
*Brassica juncea*	*BjCdR15*	Root, leaves	Nucleus	Regulate Cd uptake by roots	/	(Farinati et al., 2010)
*Tamarix hispida*	*ThbZIP1*	Leaves, stem, root	/	/	/	(Wang et al., 2010)

Cd tolerance mediated by bZIP TFs has been confirmed in various model plants and food crops. For example, in *Arabidopsis*, *AtbZIP30* activates *AtGST11* gene expression, enhancing Cd tolerance under stress conditions (Kouno and Ezaki, 2013). *AtbZIP44* regulates the expression of *AtMAN7*, influencing enzyme activity and mannose content in the cell wall, key for compartmentalization (Wu et al., 2023b). Similarly, in ramie, *BnbZIP2* overexpressing transgenic *Arabidopsis* showed higher sensitivity to the heavy metal Cd stress during seed germination, and *BnbZIP2* may play a positive regulatory role in the plant response to Cd stress (Huang et al., 2016). In *Sedum plumbizincicola*, *SpbZIP60* may enhance the tolerance of transgenic *Arabidopsis* plants to Cd by regulating ROS accumulation and protecting photosynthetic organs (Lu et al., 2022). In *T. hispida*, *ThbZIP1* is upregulated under Cd stress, enhancing Cd tolerance through the regulation of SOD activity (Wang et al., 2010). Additionally, the expression of *BpbZIP1* can be induced in *B. papyrifera* by Cd treatment; furthermore, it significantly improves Cd resistance in yeast. These findings indicate that the expression of *BpbZIP1* can improve Cd tolerance and is an important gene in the Cd stress response (Chen et al., 2022). Furthermore, the expression of the transcription factor *NtbZIP60* in *Nicotiana tabacum* is upregulated under Cd stress; hence, this gene may be a candidate gene to screen for Cd stress tolerance (Xu et al., 2013). Consistently, overexpression of *BjCdR15* (a bZIP-type TF) in *Brassica juncea*, *Arabidopsis*, tobacco enhanced Cd tolerance, and *BjCdR15* replaced the function of *TGA3* and participated in the long-distance transport of Cd from the roots to the stem; moreover, results indicated that the *BjCdR15* TF plays a crucial role in regulating Cd uptake by roots and in long-distance root-to-stem transport (Farinati et al., 2010).

Transcriptome analysis revealed that 15 bZIP TF genes responded to Cd stress in ramie, with 14 upregulated and one downregulated (Zheng et al., 2016). In soybeans, genes encoding *bZIP44* and *bZIP78* are expressed at low levels under Cd stress, indicating that they respond to Cd stress (Chmielowska-Bak et al., 2013). In rice, *OsbZIP18* and *OsbZIP23* are upregulated by Cd stress (Ailing et al., 2020). Similarly, in *Populus simonii* × *P. nigra*, *OsbZIP18* and *OsbZIP23* are upregulated under Cd stress. In total, 13 genes of the bZIP TF family are upregulated and 19 are downregulated under Cd stress (Yao et al., 2019). Furthermore, in creeping bentgrass, *bZIP06*, *bZIP43*, and *bZIP19* of the bZIP TFs are upregulated in response to Cd stress (Yuan et al., 2018). Lastly, two genes encoding bZIP (*OS06G0614100* and *OS02G0194900*) were downregulated in response to Cd stress. Hence, these bZIP TF family members may be directly involved in the response of rice plants to Cd stress (Sun et al., 2019).

Current research on bZIP TFs’ role in regulating Cd tolerance lacks detail, but evidence suggests these factors impact Cd’s long-distance transport by influencing compartmentalization and the redox system.

## Other TFs regulate Cd tolerance in plant through various pathways

Beyond the five transcription factor families-WRKY, ERF, MYB, bHLH, and bZIP-other factors involved in Cd stress regulation in plants have also gained significant attention. For example, in *S. alfredii*, *SaHsfA4c* increases plant resistance to stress by upregulating the activities of ROS-scavenging enzymes and the expression of Hsps (Chen et al., 2020). *OsNAC15* reduces Cd toxicity in rice during the vegetative period (Zhan et al., 2022). Overexpression of *TaNAC22* in wheat increases the efficiency of the enzymatic antioxidant defense system, consequently boosting Cd tolerance by regulating the expression of stress-responsive genes (Yu and Zhang, 2023). In *Arabidopsis*,*AtNAC102* regulates cell wall pectin metabolism and inhibits its binding to Cd, reducing Cd uptake and accumulation (Han et al., 2023). *ZAT6* positively regulates Cd tolerance through a glutathione-dependent pathway (Chen et al., 2016). *ZAT10* represses the transcriptional activity of *IRT1*, which encodes a key metal transporter involved in Cd uptake (Dang et al., 2022). In summary, the extensive role of transcription factors in regulating Cd has been well-documented.

## Mechanisms underlying TF regulation of *Arabidopsis* responses to Cd stress

**Figure 1:**
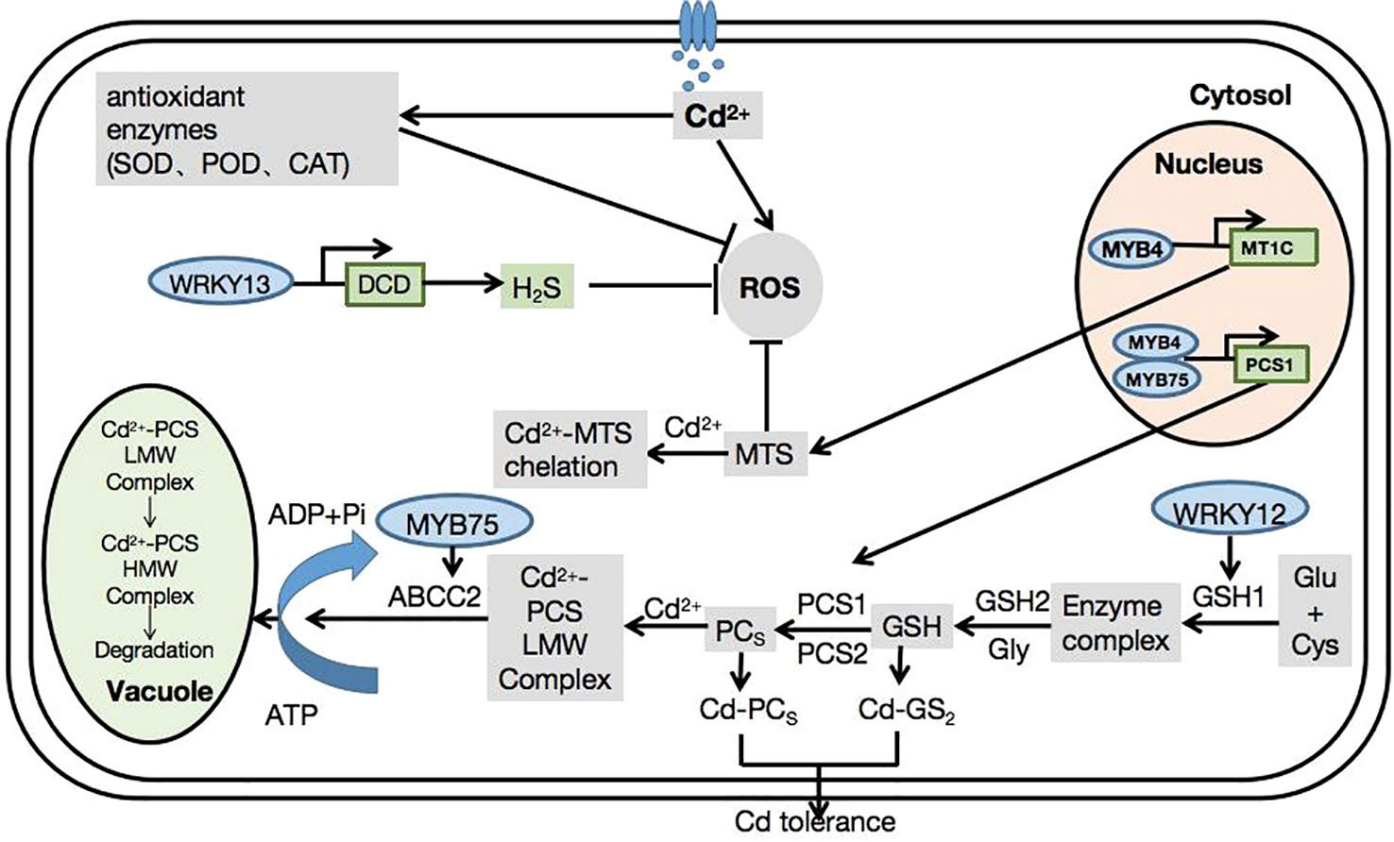
Mechanisms of transcription factor families in *Arabidopsis thaliana* in response to cadmium stress.

*Arabidopsis*, as a model plant, has made significant contributions to the study of plant biology. Recently, numerous studies have explored the mechanisms of Cd tolerance in *Arabidopsis*. However, research into the mechanisms of TF-mediated Cd tolerance and their specific functions in *Arabidopsis* remains limited. Here, we summarize the relationship between transcription factors and Cd tolerance in *Arabidopsis*. Transcription factors can regulate downstream genes that are important for Cd tolerance in plants (**Figure 1**).

In *Arabidopsis*, TFs regulate Cd accumulation via several signaling pathways. For example, *AtWRKY12* combines with the W-box of the *GSH1* promoter to directly target *GSH1*, inhibit the expression of *GSH1*, and indirectly inhibit the expression of genes related to PC synthesis (Han et al., 2019). *AtWRKY13* activates DCD expression and increases the production of H_2_S by directly binding to the DCD promoter (Zhang et al., 2020). Furthermore, it binds to the W-box region of the *PDR8* promoter, which acts downstream of *AtWRKY13*, thereby positively regulating Cd tolerance in *Arabidopsis* through PDR8 (Sheng et al., 2019). Similarly, *WRKY18*, *WRKY40*, and *WRKY60* mainly act as inhibitors of the transcription of genes encoded by H_2_S synthases (Liu et al., 2015). *ERF34* and *ERF35* bind to the promoter of the nitrate transporter *NRT1.8* (one of the transporters induced by Cd) to regulate Cd tolerance in plants (Xie et al., 2021). *ERF1B* and *ERF104* directly bind to the *NRT1.8* promoter and mediate the expression of the *NRT1.8* transporter under the action of Cd. Therefore, the regulation of plant responses to Cd stress is of great significance (Zhang et al., 2014). *AtMYB49* positively regulated the expression of *bHLH38* and *bHLH101* by directly binding to their promoters, leading to the activation of Fe-regulated *TRANSPORTER1*, which encodes a metal transporter associated with Cd uptake. *MYB49* also binds to *HIPP22* and the promoter region of *HIPP44*, leading to the upregulation of their expression (Zhang et al., 2019). *AtMYB75* acts as a positive regulator of Cd tolerance by targeting the *Arabidopsis* Cd stress-related genes *ACBP2* and *ABCC2* (Zheng et al., 2022). *AtMYB4* participates in the glutathione pathway through the transcriptional activation of *PCS1* and *MT1C*, mediating Cd tolerance through enhanced antioxidant activity (Agarwal et al., 2020). *bHLH104* positively regulates four Cd tolerance-related genes (*IREG2, MTP3, HMA3*, and *NAS4*) involved in heavy metal detoxification (Yao et al., 2018). Furthermore, *AtbZIP30* activates *AtGST11* gene expression and improves Cd tolerance in plants grown under Cd-stress conditions (Kouno and Ezaki, 2013).

Cd toxicity can be alleviated by maintaining high iron content under Cd stress. Thus, *bHLH39* and *bHLH104* functionally interact with *IMA1* and *IMA3* to enhance iron accumulation in plants by inducing the expression of iron transport genes. This leads to an increased Cd tolerance (Meng et al., 2022). Overexpression of *FIT*, *AtbHLH38*, and *AtbHLH39* activated the expression of *HMA3*, *MTP3*, *IRT2*, and *IREG2* in *Arabidopsis*, all of which are related to heavy metal detoxification. In addition, co-overexpression enhanced the expression of nicotinamine synthase (*NAS1*) and *NAS2*, leading to the accumulation of nicotinamine, a key chelating agent for iron transport and homeostasis (Wu et al., 2012).

## Mechanisms underlying TF regulation of rice and wheat responses to Cd stress

Food crop safety is closely related to human health. Rice and wheat are common food crops that enter the human body through the daily diet. If rice and wheat are grown in soils contaminated with Cd, it is highly likely that Cd would enter the human body through the food chain. Therefore, studying the mechanism of Cd stress regulation in these plants is highly necessary to provide a solid theoretical basis for further regulation of the mechanism of Cd accumulation in grain crops using molecular genetic engineering.

The *TaWRKY22* TF combined with the *TaCOPT3D* promoter directly targets *TaCOPT3D* under Cd stress, thus regulating the expression of *TaCOPT3D* and playing an important role in the regulation of Cd stress in wheat (Liu et al., 2022a). *TaWRKY70* transgenic *Arabidopsis* showed increased SOD, POD, and CAT activities under Cd stress. Therefore, *TaWRKY70* regulates Cd stress by improving plant antioxidant capacity (Jia et al., 2021). *TaWRKY74* affects the absorption and transport of Cd in wheat by affecting the expression of ASA-GSH synthesis genes and inhibiting the expression of Cd transporter genes (Li et al., 2022). In contrast, *TaMYC8* activates *TaERF6* transcription by binding to the *TaERF6* promoter to directly regulate the expression of *TaERF6*, thus negatively regulating Cd tolerance (Wang et al., 2022b). *TdSHN1* mutant tobacco exhibits higher ROS-scavenging enzyme (SOD and CAT) activity. This might explain why transgenic tobacco plants are more resistant to heavy metals (Djemal and Khoudi, 2022).

In rice, the expression level of osa-miR5493 is negatively correlated with that of *OsERF141*. This finding implies that osa-miR5493 may also be a key regulator of Cd stress through the regulation of *OsERF141* (Ailing et al., 2020). *OsTAZ4* promotes plant resistance to Cd stress by regulating ROS homeostasis and can promote plant resistance to various heavy metals by interacting with the *OsMYB34* (Shalmani et al., 2021). In the *OsMYB36* knockout mutant, Cd accumulation decreased, and the expression of *OsNramp5* was significantly altered, indirectly indicating that *OsMYB36* may affect rice Cd tolerance by regulating the expression of *OsNramp5* (Hu et al., 2017).

## Conclusions

The five major transcription factor families in plants-WRKY, ERF, MYB, bHLH, and bZIP-are primarily located in the nucleus and positively respond to Cd stress. Although expressed in all plant tissues, their expression is predominantly in the roots. The WRKY transcription factor family mediates Cd tolerance by regulating the H_2_S signaling pathway, altering ROS levels, and modulating antioxidant enzyme activities. The ERF transcription factor family regulates Cd tolerance through processes that influence ROS, nitrate, and ethylene synthesis and activity. The MYB transcription factor family regulates the synthesis and activity of antioxidant enzymes, facilitating Cd compartmentalization. The bHLH transcription factor family plays a crucial role in Cd transport from roots to stems and enhances iron accumulation by inducing iron transport gene expression. The bZIP transcription factor family is critical for regulating Cd uptake by roots and its long-distance transport from root to stem. The WRKY, ERF, MYB, bHLH, and bZIP TFs are crucial in regulating Cd tolerance in both model plants and food crops. Therefore, it is essential to study the mechanisms through which these families regulate plant tolerance to Cd.

Although the mechanisms of Cd tolerance in plants are well understood, the specific roles of transcription factors are not thoroughly explored, with limited studies available, Therefore, further investigation of the function of TFs in Cd tolerance in plants, particularly in model plants and food crops, is necessary. A thorough understanding of the molecular mechanisms underlying Cd tolerance in plants can be utilized for genetic engineering. Biotechnology is used to alter the genetic characteristics of organisms from a genetic point of view. This aims to enable the insertion, removal, and replacement of genes of interest, which often play important roles in the regulation of biotic and abiotic stress resistance. Through such purposeful alterations, precise regulation can be achieved to obtain desired plant traits. Therefore, genetic engineering is an effective means to improve biotic and abiotic stress resistance in plants, including that to heavy metals. To appropriately modify plants using genetic engineering, the basic functions of plant genes must be understood.

## Author contributions

HZ: Formal analysis, Visualization, Writing – original draft. LL: Funding acquisition, Supervision, Writing – review & editing.
